# Antenatal Pathology and Hygiene: The Embryo

**Published:** 1907-09

**Authors:** 


**Antenatal Pathology and Hygiene: the Embryo.** By J. W. Ballantyne, M.D. Pp. xix., 697. Edinburgh: William Green and Sons. 1904.

This volume is a most interesting and exhaustive statement of the results of the author's researches in a domain of pathology hitherto insufficiently subjected to scientific study. No pains have been spared to obtain the fullest possible information on the subject, and numerous specimens of various types of fœtal monstrosities and instances of congenital defects and abnormalities for the purpose of scientific description and classification. It is difficult in dealing with such a work to decide on the parts of it to which attention should be directed. While it is necessary that the whole volume should be read connectedly, in order that the valuable information contained in it may not be missed, we may still direct attention to portions of it which are perhaps of rather more general interest than others. A most detailed and clear account of the embryology is, with its chronology, given to commence with, but, as the author very justly remarks, as regards human embryology our knowledge practically commences with the early ova described by Hubert Peters and Leopold.

We know nothing practically of the changes which occur in the fertilised human ovum before the fifth day after impregnation, which is the approximate date of the Peter's ovum, that of Leopold being a little older.

Capital plates of sections of these two ova are introduced, but unfortunately the reference lettering is not always as distinct as it might be. One of the most interesting sections is the account of experimental terato-genesis, detailing the experiments of the St. Hilaires, Charles Féré and C. Darester on the incubation of hens' eggs, showing how in various ways, by mechanical interference with the developing ovum or by subjecting it to various toxic or unnatural thermal or other influences, it is possible to produce various types of monstrous chicks.

He suggests that monsters are produced invariably by the arrest of development of a part of the fœtus at a certain stage, while the remaining parts progress normally, and shows that many monstrosities are represented at certain stages of normal development.

The chapter on maternal impressions should also be read carefully by those interested. He gives a most interesting historical account of these so-called phenomena, and one or two curious instances of his own researches into the origin of histories supposed to be established as scientific facts. When a scientific writer attributes to a certain observer as a fact observed and recorded by him a statement which was to the effect that he had seen the record made by another observer, it is easy to understand how, among the uninstructed public in any country, the extraordinary stories, many of which he quotes, become current and obtain universal acceptance. The account of the pathology of hydatid mole is extremely interesting, and explains many of the more intricate features of the pathology of that disease.

As we have already stated, it is extremely difficult to select sections of a work of this kind; it must be read as a whole, and will amply repay the labour of perusal by anyone interested in the subject. It is an excellent presentation in a truly scientific form of facts hitherto isolated and often exaggerated in description, classifying all the abnormalities described in a coherent and rational manner, and giving the author's deductions from facts observed by himself, or accurately recorded by others, in a manner at once lucid and forcible.

